# Pictorial essay: MRI of the fetal brain

**DOI:** 10.4103/0971-3026.45349

**Published:** 2009-02

**Authors:** Ganesh Rao B, BS Ramamurthy

**Affiliations:** Ragavs Diagnostic and Research Centre, Sadguru Complex, 27^th^ Cross, 4^th^ Block, West, Jayanagar, Bangalore- 560 011, India; 1 Srinivasa Ultrasound Scanning Centre, 48/2, Shankar Mutt Road, Shankarpuram, Bangalore - 560 004, India

**Keywords:** Brain, fetal, MRI

## Abstract

MRI is a useful supplement to USG for the assessment of fetal brain malformations. Superior soft tissue contrast and the ability to depict sulcation and myelination are the strengths of MRI. Subtle or inconclusive USG abnormalities can be confirmed or ruled out by MRI. In some cases, additional findings detected with MRI often help in arriving at a definitive diagnosis, which is necessary for parental counseling and for guiding management. Fast T2W sequences form the basis of fetal MRI. There have been no reports of deleterious effects of MRI on the fetus. A few case examples are presented to illustrate the advantages of MRI.

## Introduction

USG is the primary modality used to assess the fetus. USG examination by a skilled operator, in most cases, provides adequate information regarding fetal morphology, its environment, and its well-being. The quality of USG, however, is adversely affected by factors such as maternal obesity, unfavorable fetal position, multiple gestation, decreased amniotic fluid, or the near-field reverberation artifact (relevant for fetal cranial imaging) [Table 1]. The abnormalities detected on USG may at times be very subtle or inconclusive. In such cases, several studies have shown that MRI is a helpful modality.[1–3] In the context of the fetal brain, some aspects such as maturation and myelination can only be assessed by MRI.[4,5] This essay describes the ideal timing of the MRI examination, safety issues, technique and various indications illustrated and explained by typical examples and cases.

**Table 1:** Comparison USG vs. MRI

Feature	US	MRI
Multiplanar	✓	✓
Field of View	✗	✓
Soft Tissue Contrast	✗	✓
Obesity	✗	✓
Oligohyramnios	✗	✓
Foetal position	✗	✓
Near half resolution	✗	✓
Foetal movements	✓	✗
Claustrophobia	✓	✗
Cost, availability	✓	✗
Spatial Resolution	✓	✗

✓-has an advantage, ✗-Is at a disadvantage

## Timing of the MRI study

The need for early prenatal diagnosis, parental anxiety (after a suspicious finding on USG), and local legislation[6] regulating termination of pregnancy are the factors which necessitate early MRI examination of the fetus.

It is relevant to note that the corpus callosum and the vermis are completely formed by the end of 18 weeks of gestation. Fetal brain sulcation begins at around the same gestational age. When deciding on the timing of MRI, one needs to consider the natural history of the fetal disorder in question. For example, in the case of fetal cytomegalovirus infection and tuberous sclerosis, the cranial findings may become apparent only in the third trimester and MRI done too early may do more harm than good. Since most USG abnormalities are generally detected in an anomaly scan done at around 20 weeks of gestation, most cases are referred for MRI around the same time.

## Safety issues

Many studies have shown that fetal MRI examination is not associated with any major deleterious effects.[7] No health risks have been reported at field strength of 1.5-Tesla (1.5-T). No adverse outcomes have been observed in pregnant MRI workers.[8] The Safety Committee of the Society for MRI has concluded that prenatal MRI is indicated when other nonionizing diagnostic imaging methods are inadequate or when MRI examination can provide important information that would otherwise require the use of ionizing radiation.[9] There is currently no data regarding the level of acoustic noise experienced by the fetus during the MRI procedure.[10,11] The use of gadolinium is not recommended because of the need to avoid potential deleterious effects on the fetus.[12–14] The National Radiation Protection Board and the Food and Drug Administration have approved MRI only after the first trimester.[15,16] The safety of the newer techniques of diffusion-weighted imaging (DWI), diffusion tensor imaging (DTI), MRI spectroscopy (MRS), and functional MRI (F-MRI) has not yet been proven.[17]

## MRI technique

Informed consent from the mother has to be obtained before the procedure. Fetal MRI is performed on a high-field strength MRI scanner (1.5-T); a phased-array surface coil is placed over the abdomen and pelvis, with the patient in the supine or lateral decubitus position. Polyhydramnios and multiple gestations may increase the distance between the region of interest and the surface coil, resulting in a reduction in the signal strength.[18,19] Maternal premedication has been used to achieve fetal sedation, although the fast sequences available on the newer scanners obviate the need for sedation. Maternal breathholding during sequences is desirable.[5,20]

**Figure 1 (A,B):**
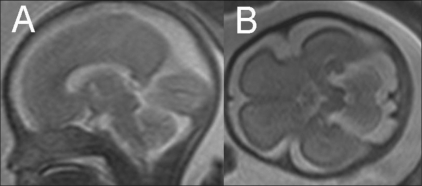
T2W sagittal MRI image (B) at 27 weeks shows the parieto-occipital sulcus (arrowhead), calcarine sulcus (open arrow), and primary cerebellar fissure (closed arrow). A T2W axial MRI image (B) shows myelination (hypointense region) in the posterior brainstem (arrowhead)

**Figure 2 (A,B):**
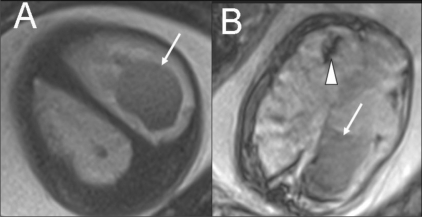
Single live gestation at 33 weeks. T2W (A) and gradient-echo T2W (T2*) MRI images show intraventricular (arrows) and periventricular (arrowhead) hemorrhage. Fetal blood sampling was negative for TORCH IgMs. A platelet count of 50,000 / mm3 was noted. Pregnancy ended with a stillbirth at 35 weeks.

**Figure 3:**
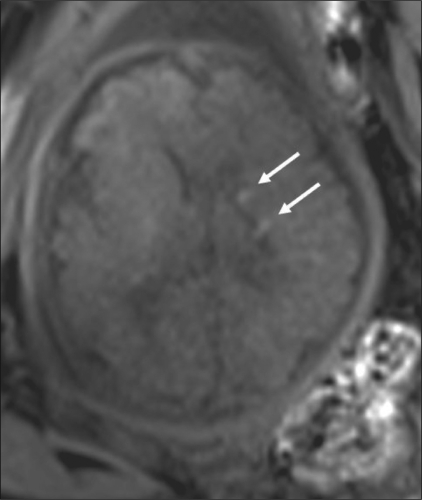
Single live gestation at 30 weeks. T1W axial MRI image at the level of the lateral ventricles shows tubers as subependymal hyperintense nodules (arrows).

Fetal motion significantly degrades image quality. For this reason, fast T2W single-shot imaging techniques such as single-shot fast spin-echo (SSFSE), half-Fourier acquisition turbo spin-echo (HASTE), and rapid acquisition with relaxation enhancement (RARE) have been the most widely used pulse sequences for fetal MRI.[21–24] Such fast acquisition sequences provide good contrast and spatial resolution and are suitable for surface delineation, sulcational analysis, and biometry [Figure 1]. Studies mention a lag of one week of the brain development compared to the neuroanatomic findings.[25] Gradient-echo T2W images (T2*) are accurate in the detection of chronic hemorrhagic lesions and calcifications[5] [Figure 2]. T1W images are well suited for demonstrating small hyperintense lesions like tubers, calcification, lipomas, and laminar necrosis[21,26–29] [Figure 3]. There are reports which mention a good correlation between USG and MRI measurements of biparietal diameter, head circumference and cerebellar width.[30–32] An initial fast localizing sequence is performed; sequences are then acquired in orthogonal planes relative to the immediately preceding plane. Imaging planes are chosen to represent sections relative to the fetus.[5,19]

We normally use the following sequences [Table 2] on our 1.5-T MRI (Avanto, Siemens, Erlangen, Germany) unit:

**Table 2:** Sequences

Sequence	TR	TE	Flip angle	FOV	BW	Slice thickness	Distance factor	Dimension
T2 HASTE	1000	120	144	300	400	2-4 mm	10%	2D echo
T1 Fl 2D	120	5	70	370	120	3-6 mm	10%	2D echo
T2 trufi	4.07	2.04	58	350	500	2-4 mm	10%	2D echo

## Indications and case studies

MRI is indicated in situations when a brain anomaly has been detected on USG, but the diagnosis is not obvious or definite and needs confirmation.

**Figure 4 (A-D):**
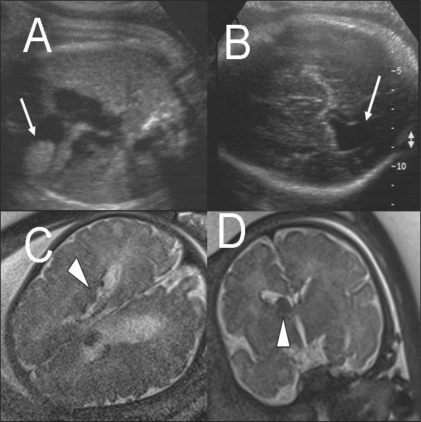
Single live gestation at 37 weeks. USG scans of the heart (A) and brain (B) show a cardiac rhabdomyoma (arrow in A) and mild ventriculomegaly (arrow in B). Axial (C) and coronal (D) T2W MRI images show subependymal tubers (arrowheads), confirming the diagnosis of tuberous sclerosis. The large left lateral ventricular subependymal nodule at the foramen of Monroe is probably responsible for the unilateral ventriculomegaly. The infant developed ash-leaf macules at 1 month and myoclonic jerks at 2 months of age. It died at 3 months (cradle death)

**Figure 5 (A-F):**
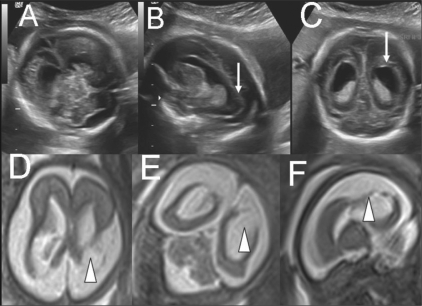
Single live gestation at 24 weeks. USG images (A-C) in different planes show ventriculomegaly with prominent occipital horns (arrows) and widened subarachnoid spaces. The possibilities of neuronal migrational and an atrophic disorder were considered. T2W axial (D), coronal (E), and sagittal (F) MRI images show bilateral symmetric total neuroparenchymal loss in the parieto-occipital watershed regions (arrowheads) but no clefting; this is suggestive of an ischemic etiopathogenesis rather than a neuronal migrational disorder. The couple opted for termination of pregnancy. They declined an autopsy

**Figure 6 (A-D):**
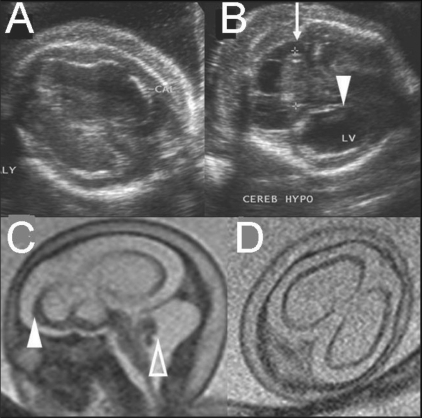
Single live gestation at 25 weeks. USG images (A,B) show cortical atrophy (A) and cerebellar hypoplasia (arrow in B), ventriculomegaly, and periventricular calcification (arrowhead in B). Sagittal T2W MRI image (C) shows marked cortical thinning (solid arrowhead) and marked cerebellar hypoplasia (open arrowhead) in a suspected case of fetal CMV infection. Axial T2W MRI image (D) fails to demonstrate calcification. The couple declined prenatal testing and opted for termination of pregnancy.

Marginal ventriculomegaly is one such instance. MRI may detect the presence of heterotopia or sulcational disorders, which have profound prognostic and counseling implications. In the case illustrated in Figure 4, the presence of fetal cardiac rhabdomyoma and unilateral marginal ventriculomegaly prompted the MRI examination. Multiple subependymal tubers were demonstrated on MRI, thus clinching the diagnosis of tuberous sclerosis. Bilateral ventriculomegaly, especially of the occipital horns, was the indication for MRI in the case illustrated in Figure 5. Here, MRI clearly demonstrates bilateral, symmetric, neuroparenchymal loss in the parieto-occipital watershed regions as against the USG possibility of clefting; these findings suggest an ischemic etiopathogenesis rather than a neuronal migrational disorder such as schizencephaly. MRI demonstrated the extent, location, symmetry, and morphology of the defects better than USG. In the case illustrated in Figure 6, USG detection of ventriculomegaly, periventricular calcification, cortical thinning, and cerebellar hypoplasia led to a referral for MRI. MRI confirmed the severity of cerebral and cerebellar atrophy but failed to demonstrate calcification. Congenital cytomegalovirus infection was then considered.

**Figure 7 (A-D):**
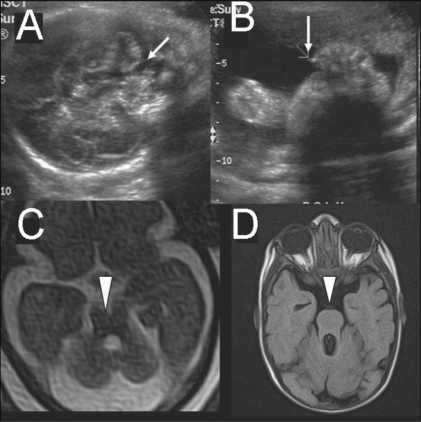
Single live gestation at 28 weeks. USG images (A,B) show inferior vermian agenesis (arrow in A) and postaxial polydactyly (arrow in B). T1W axial image (C) does not show the ‘molar tooth sign’ (arrowhead). Postnatal T1W axial MRI image at 4 months shows the ‘molar tooth sign’ (arrowhead), confirming the diagnosis of Joubert's syndrome. In this case, the mother noticed abnormal eye movements and failure of fixation of vision by the infant at 3 months of age

Uncertain findings on USG can by supplemented by an MRI examination. In the case described in Figure 7, USG detected inferior vermian agenesis and postaxial polydactyly. Fetal MRI was done specifically to look for the ‘molar tooth sign’ which, however, was not seen. Postnatally, at 3 months of age, this infant presented with the clinical features of Joubert's syndrome. A repeat MRI demonstrated the presence of the ‘molar tooth sign,’ thus confirming the diagnosis of Joubert's syndrome. This is an instance of a disease that evolved with the passage of time.

**Figure 8 (A-D):**
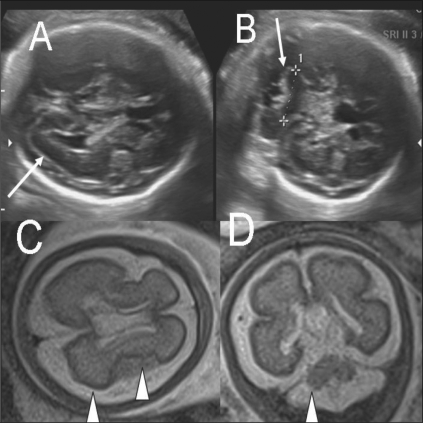
Single live gestation at 31 weeks. USG images (A,B) show suspected cortical atrophy (arrow in A) and cerebellar hypoplasia (arrow in B). Axial T2W HASTE MRI image (C) shows poor sulcation and shallow Sylvian fissures with poor opercularization (arrowheads), diagnostic of lissencephaly. Coronal T2W MRI image (D) shows cerebellar hypoplasia (arrowhead). This case was lost to follow-up

In Figure 8, cerebellar hypoplasia & suspected cortical atrophy at 31 weeks gestational age were the clues on USG. MRI demonstrated poor sulcation, poor opercularization, and shallow Sylvian fissures, confirming lissencephaly.

**Figure 9 (A-G):**
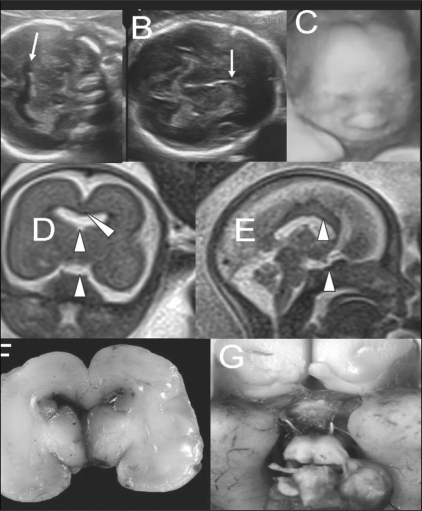
Pregestational diabetic mother with a 20 weeks' single live gestation. USG images (A,B) show fusion of the frontal horns of the lateral ventricles (arrow in A) and absent cavum septum pellucidum (arrow in B), indicating callosal agenesis. 3-D USG image (C) shows a normal fetal face. T2W coronal MRI image (D) shows absent septum pellucidum, midline cerebral fusion (solid arrowhead), and indistinct optic chiasm (open arrowhead). T2W sagittal MRI image shows the presence of a corpus callosum (open arrowhead) but a possibly hypoplastic hypophysis (solid arrowhead). Autopsy (F,G) images show frontal horn fusion, absent septum pellucidum, and a small optic chiasm, confirming the diagnosis of septo-optic dysplasia

In some instances, overt intracranial findings on USG may necessitate MRI examination as a second confirmatory tool. The example of a case of USG-detected frontal horn fusion with agenesis of septum pellucidum is illustrated in Figure 9. Here, MRI confirmed the USG findings and also revealed an indistinct optic chiasma and possible hypophyseal hypoplasia. This clinched the diagnosis of septo-optic dysplasia.

MRI can confirm callosal agenesis or dysgenesis in cases suspected by USG. Additionally, MRI may also demonstrate heterotopia. USG-detected fetal intracranial tumors can be characterized by MRI^34^. When USG is unable to differentiate between fetal intracranial bleed and tumor, MRI can help resolve the issue.[35]

Fetal MRI is increasingly being used as a means of surveillance in situations where fetal brain lesions are anticipated. An example of such a situation is the monitoring of the surviving twin after the co-twin's demise in monochorionic twinning.[36] MRI is also indicated when there is a history of a previous child with a genetic disorder, e.g., Miller-Dieker syndrome.[37]

MRI examination of the fetal brain requires extensive training. As with USG examination, an MRI examination should also be performed in a systematic manner. The USG findings and the history should be known to the radiologist. It is best if the radiologist performing the MRI is provided with the questions that have to be specifically answered. The systematic approach typically includes biometry, maturational / sulcational analysis, and myelination analysis.[30–33] The ability to accurately measure cerebral and bone biparietal diameters enables quantification of any fetal cerebral neuroparenchymal loss. Callosal length and vermian height and area are measurements that MRI can provide with ease, though with the advent of three-dimensional studies these measurements may also be assessed by USG. A detailed discussion of sulcation and myelination analysis is beyond the scope of this article.

Newer techniques such as DWI, DTI, MRS, and F-MRI are being tried out. DWI may be useful to study hypoxia and ischemia affecting the fetal brain[36] and DTI for studying the development of neural tracts like the corpus callosum, the optic tracts, and the anterior commisure.[38] MRS has the potential to be useful in the evaluation of myelination, ischemia, hemorrhage, metabolic variations in brain damage and inborn errors of metabolism.[39]

Fetal MRI is a powerful supplement to USG and enables us to demonstrate findings that cannot be recognized on USG. Appropriate integration of fetal MRI into the prenatal evaluation algorithm can improve decision-making and patient care. Clinching of a diagnosis enables cross-specialty consultation amongst radiologists, obstetricians, neuropediatricians, and geneticists; this allows more targeted and meaningful counseling and management strategies.
